# MDA-9/Syntenin-Slug transcriptional complex promote epithelial-mesenchymal transition and invasion/metastasis in lung adenocarcinoma

**DOI:** 10.18632/oncotarget.6299

**Published:** 2015-11-02

**Authors:** Lu-Kai Wang, Szu-Hua Pan, Yih-Leong Chang, Pei-Fang Hung, Shih-Han Kao, Wen-Lung Wang, Ching-Wen Lin, Shuenn-Chen Yang, Chen-Hsien Liang, Chen-Tu Wu, Tzu-Hung Hsiao, Tse-Ming Hong, Pan-Chyr Yang

**Affiliations:** ^1^ Department of Internal Medicine, College of Medicine, National Taiwan University, Taipei, Taiwan; ^2^ Graduate Institute of Medical Genomics and Proteomics, College of Medicine, National Taiwan University, Taipei, Taiwan; ^3^ Doctoral Degree Program of Translational Medicine, College of Medicine, National Taiwan University, Taipei, Taiwan; ^4^ Department of Pathology and Graduate Institute of Pathology, College of Medicine, National Taiwan University, Taipei, Taiwan; ^5^ NTU Center of Genomic Medicine, College of Medicine, National Taiwan University, Taipei, Taiwan; ^6^ Research Center for Tumor Medical Science, China Medical University, Taichung, Taiwan; ^7^ Department of Otolaryngology, Kaohsiung Chang Gung Memorial Hospital, Kaohsiung, Taiwan; ^8^ Institute of Biomedical Sciences, Academia Sinica, Taipei, Taiwan; ^9^ Division of Isotope application, Institute of Nuclear Energy Research, Taoyuan, Taiwan; ^10^ Department of Medical Research, Taichung Veterans General Hospital, Taichung, Taiwan; ^11^ Institute of Basic Medical Sciences, National Cheng Kung University, Tainan, Taiwan; ^12^ Institute of Clinical Medicine, National Cheng Kung University, Tainan, Taiwan

**Keywords:** Syntenin, Slug, EMT, invasion, lung adenocarcinoma

## Abstract

Melanoma differentiation-associated gene-9 (MDA-9)/Syntenin is a novel therapeutic target because it plays critical roles in cancer progression and exosome biogenesis. Here we show that Slug, a key epithelial-mesenchymal-transition (EMT) regulator, is a MDA-9/Syntenin downstream target. Mitogen EGF stimulation increases Slug expression and MDA-9/Syntenin nuclear translocation. MDA-9/Syntenin uses its PDZ1 domain to bind with Slug, and this interaction further leads to HDAC1 recruitment, up-regulation of Slug transcriptional repressor activity, enhanced Slug-mediated EMT, and promotion of cancer invasion and metastasis. The PDZ domains and nuclear localization of MDA-9/Syntenin are both required for promoting Slug-mediated cancer invasion. Clinically, patients with high MDA-9/Syntenin and high Slug expressions were associated with poor overall survival compared to those with low expression in lung adenocarcinomas. Our findings provide evidence that MDA-9/Syntenin acts as a pivotal adaptor of Slug and it transcriptionally enhances Slug-mediated EMT to promote cancer invasion and metastasis.

## INTRODUCTION

Lung cancer mortality, especially in non-small cell lung cancers (NSCLCs), remains the leading healthcare issue in cancer therapy worldwide [1, 2] and metastasis is the major cause of treatment failure [2–4]. The EMT, the crucial initiating step of metastasis, is regulated by a group of transcription repressors, including zinc finger proteins such as the Snail, or Zeb family proteins [5]. Slug, a member of the Snail family, participates in many physiologic processes, including mesoderm formation, cell migration and invasion [6], and Slug expression correlates with clinical outcome in NSCLC [7–9].

Slug proteins are maintained at low levels in low invasive cancer cells through various mechanisms such as ubiquitin-mediated proteosomal degradation. Recent reports show that p53/MDM2 and GSK-3β/CHIP are capable of reducing cancer cell invasion by modulating Slug degradation [7, 10, 11]. Slug transcriptionally represses E-cadherin expression, thereby promoting the EMT process in development, as well as cancer invasion and metastasis [12]. However, the detailed regulatory mechanisms of Slug-mediated transcriptional control of cancer invasion remain unclear.

Melanoma differentiation-associated gene-9 (MDA-9)/Syntenin is a scaffold protein that contains two tandem PDZ domains. It is involved in a variety of cellular processes by organizing protein complexes, thereby acting as a mediator that transmits cell signaling from the plasma membranes, facilitates intracellular protein trafficking, or stimulates exosome production [13–18]. Recent studies indicate that up-regulation of MDA-9/Syntenin may lead to cancer progression by binding to kinases or receptor on the plasma membrane and mediating the activation of P38/NF-κB, Smad signaling or the AKT pathway in several types of cancer [19–28]. Moreover, several studies indicate that MDA-9/Syntenin may also exist in the nucleus of highly invasive cancer cells and correlate with cancer progression [29, 30]. However, the function of nuclear MDA-9/Syntenin needs further investigation.

This study revealed the molecular mechanisms involved in the regulation of Slug function and control of cancer cell invasion/metastasis by MDA-9/Syntenin. Moreover, EGF stimulation can enhance nuclear translocation of MDA-9/Syntenin, and the nuclear MDA-9/Syntenin interacts binds with Slug to form the transcriptional repression complex within the nucleus, enhances Slug transcriptional repressor activities, and promotes EMT, invasion, and metastasis in lung adenocarcinoma.

## RESULTS

### MDA-9/Syntenin interacts with Slug and is co-localized in the nucleus

**Figure 1:**
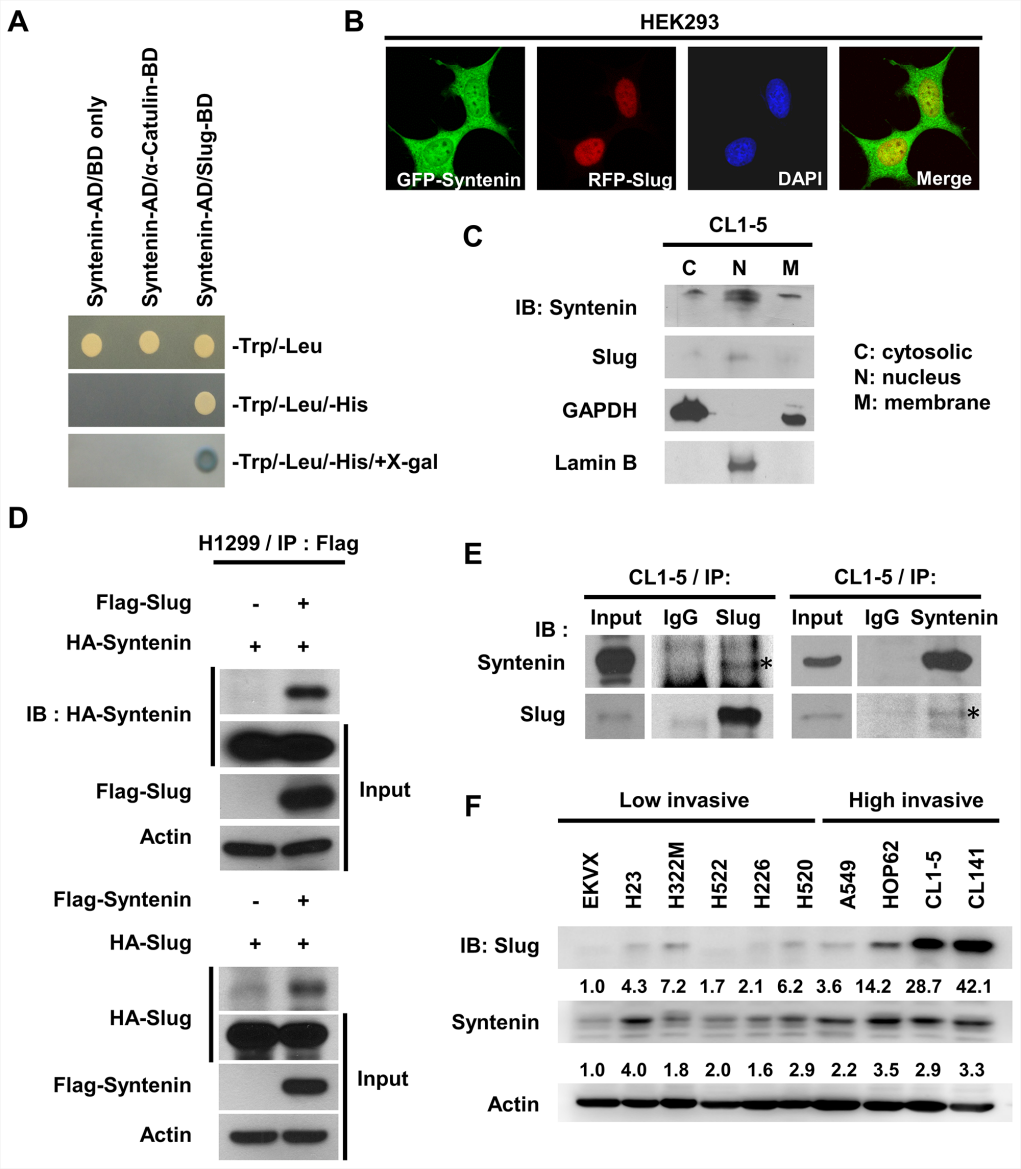
MDA-9/Syntenin was associated with Slug and its expression level positively correlated with invasive capacity in lung adenocarcinoma cell lines **A.** Binding of MDA-9/Syntenin to Slug in the yeast two-hybrid assay. Selected colonies of pACT2-MDA-9/Syntenin with constructs in pAS2 vector were grown on selection medium. Vector and α-catulin were used as negative control. AD, activating domain; BD, binding domain. **B.** Confocal images of HEK293 cells transiently transfected with pEGFP-Syntenin and pDsRed-Slug. **C.** Western blot analysis of endogenous MDA-9/Syntenin and Slug protein distribution in CL1–5 cells. Lamin B and GADPH were indicators for the nucleus and cytosol fractions, respectively. **D.** Immuno-precipitation and Western blot analysis of MDA-9/Syntenin interaction with Slug by transiently transfecting the indicated plasmids in H1299 cells or **E.** at the endogenous level in CL1–5 cells. The input panel shows the 1/20 of whole-cell lysates used for Immuno-precipitation. **F.** Lung adenocarcinoma cell lines were lysed and Slug and MDA-9/Syntenin protein expressions were analyzed directly by immunoblotting.

To identify the regulatory mechanism of Slug in cancer invasion, the yeast-two hybrid system was used to screen for the potential association proteins of Slug (Figure S1A). MDA-9/Syntenin was found in the pool. The β-gal assay further confirmed that MDA-9/Syntenin interacted with Slug (Figure 1A). To examine the subcellular distributions of Slug and MDA-9/Syntenin and their interaction, GFP-tagged Syntenin and DsRED-tagged Slug fusion proteins co-transfected into HEK293 cells were used. The results suggested that GFP-tagged Syntenin was partially co-localized with DsRED-tagged Slug protein in the nucleus (Figure 1B). To examine their expressions, CL1–5 cells were fractionated, and both Slug and MDA-9/Syntenin co-existed in the nuclear fraction in CL1–5 cell line (Figure 1C).

Both over-expression and endogenous co-immunoprecipitation assays were performed to confirm the interaction of MDA-9/Syntenin and Slug in lung adenocarcinoma cell lines. The results showed that MDA-9/Syntenin and Slug proteins associated with each other when they were both overexpressed in H1299 cells (Figure 1D). Moreover, the endogenous interaction between MDA-9/Syntenin and Slug were confirmed in CL1–5 cells by immunoprecipitation (Figure 1E). Endogenous expression levels of MDA-9/Syntenin and Slug in H1299, CL1–5, and CL141 were also noted (Figure S1B). Together, these data show that MDA-9/Syntenin and Slug are both localized in the nucleus and associated with each other.

Because Slug is an invasion-promoting factor and often highly expressed in aggressive cancer cells, the correlation between the expressions of these two proteins and cell invasiveness were then investigated. In a panel of several lung cancer cell lines, including EKVX, H23, H322M, H522, H520, A549, HOP62, H226, CL1–5, and CL141, both MDA-9/Syntenin and Slug expressions positively correlated with the invasiveness of lung adenocarcinoma cell lines (Figure 1F) and four breast cancer cell lines (Supplementary Figure 1C), suggesting that MDA-9/Syntenin and Slug may correlate with cell invasiveness in general.

### MDA-9/Syntenin and Slug both regulate EMT and invasiveness in lung adenocarcinoma cell lines

**Figure 2:**
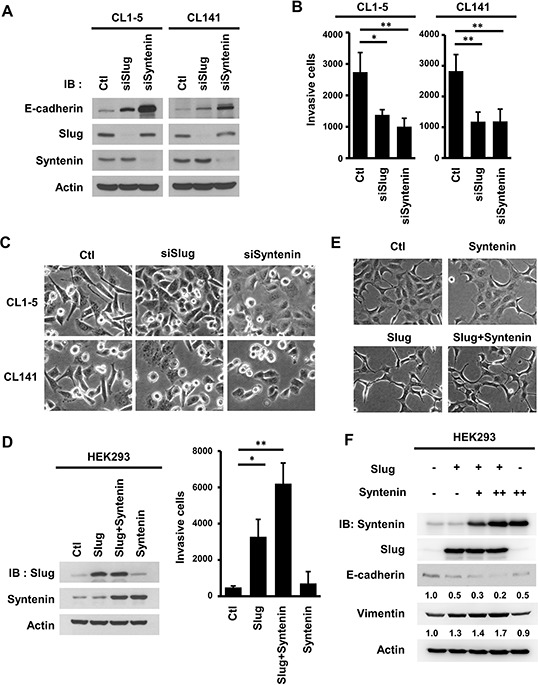
MDA-9/Syntenin enhanced Slug-mediated E-cadherin regulation and cell invasion **A.** Measurement of E-cadherin expression level, **B.** cell invasion, and **C.** cell morphology in CL1–5 and CL141 cells transiently transfected with siCtl, siSlug, or siSyntenin for 72 h. The bar graph of cell invasion shows the mean number of invasive cells ± SD (*n* = 3, *p < 0.05, **p < 0.001). **D.** Measurement of the cell invasion in HEK293 cells transiently infected with Slug- and MDA-9/Syntenin-expressing vectors. Invasive cells are represented as mean ± SD (*n* = 3, *p < 0.05, **p < 0.001). **E.** At 72 h post-lentiviral infection, the cell morphology of HEK293 cells expressed with control, Slug, MDA-9/Syntenin, or Slug+MDA-9/Syntenin were photographed by microscopy. **F.** Western blot analysis of E-cadherin and vimentin in HEK293 cells expressing control, Slug, MDA-9/Syntenin, or Slug+MDA-9/Syntenin. (+ : MOI = 1.5, ++ : MOI = 3).

To determine if MDA-9/Syntenin and Slug affected cell invasiveness in lung adenocarcinoma cell lines, their expressions were knocked down and *in vitro* invasion assays were performed. Knockdown of either siRNAs or lentivirus-transduced shRNAs specifically targeting against MDA-9/Syntenin or Slug increased E-cadherin expressions (Figure 2A and Supplementary Figure 2A). Furthermore, a typical mesenchymal marker, N-cadherin, was suppressed in Slug or MDA-9/Syntenin-silenced CL1–5 and CL141 stable cells, indicating that these cells had undergone EMT.

Silencing Slug or MDA-9/Syntenin expression in highly invasive cells decreased cancer cell invasiveness (Figure 2B) and enhanced cell-cell adhesion (Figure 2C). Thus, both MDA-9/Syntenin and Slug can promote the EMT and cell invasiveness in highly invasive lung cancer cells.

### Overexpression of MDA-9/Syntenin enhances Slug-mediated E-cadherin suppression and cancer cell invasiveness

To study whether MDA-9/Syntenin enhances EMT and invasion through regulating Slug, the effects of cell invasive capacity, cell morphology, and the expression levels of EMT markers were examined in HEK293 cells that had low expression levels of both proteins (Supplementary Figure 1B). The results showed that overexpression of Slug and MDA-9/Syntenin alone in HEK293 cells increased cancer cell invasiveness by 6.72-fold and 1.44-fold, respectively. Co-expression of Slug and MDA-9/Syntenin markedly increased cell invasiveness by 12.72-fold (Figure 2D). Both cells overexpressing Slug alone and Slug+MDA-9/Syntenin had conspicuous morphologic changes to spindle-like shapes (Figure 2E). Increased MDA-9/Syntenin expression enhanced Slug-mediated E-cadherin suppression and elevated vimentin expression (Figure 2F). These data indicate that MDA-9/Syntenin enhances Slug-mediated E-cadherin expression and cell invasiveness.

### MDA-9/Syntenin modulates Slug-mediated cancer invasion and metastasis

**Figure 3:**
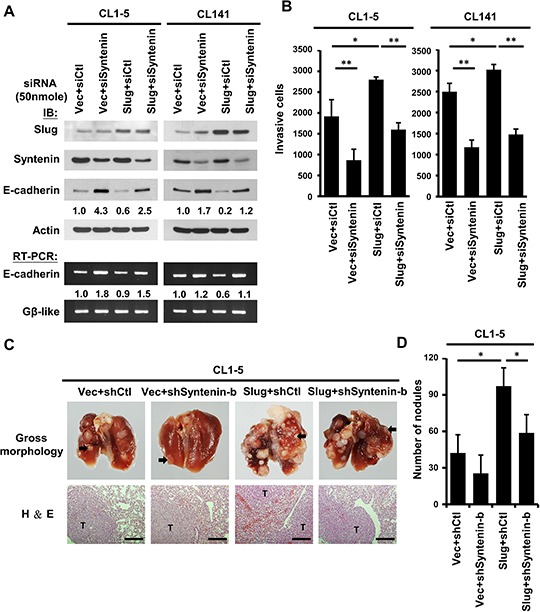
Knockdown of MDA-9/Syntenin expression decreased Slug-promoted E-cadherin suppression and cancer invasion/metastasis **A.** CL1–5 (left) and CL141 (right) cells-expressing control or Slug were transiently transfected with siCtl and siSyntenin, respectively. Protein and mRNA expression levels of E-cadherin in these cells were determined by Western blotting and RT-PCR, respectively. **B.** Invasive capacity was determined with the Boyden chambers invasion assay. Invasive cells are represented as mean ±SD (*n* = 3, *p < 0.05, **p < 0.001). **C.** Effects of MDA-9/Syntenin silencing in Slug-promoted metastasis *in vivo*. Top panel: Representative lungs of mice intravenously (i.v.) injected with CL1–5/Vector+shCtl, CL1–5/Vector+shSyntenin-b, CL1–5/Slug+shCtl, and CL1–5/Slug+shSyntenin-b stable cells (black arrows indicate lung tumors). Bottom panel: Histologic examination of lung tumors by hematoxylin-eosin (H&E) staining. Scale bars, 200 μm. **D.** Number of metastatic tumor nodules were calculated five weeks after tail-vein injection (*n* = 6 per group). Tumor nodules are represented as mean ± SEM.

To investigate whether MDA-9/Syntenin affects Slug-mediated regulation in lung adenocarcinoma cells, the expression of MDA-9/Syntenin in CL1–5 and CL141 cells was silenced to confirm the effects of MDA-9/Syntenin on the repression activities of Slug. The results indicated that E-cadherin expression was decreased in Slug-overexpressing cells compared to control cells (Figure 3A, lane 3 in CL1–5 and CL141). Decreased E-cadherin expression was reversed after the expression of MDA-9/Syntenin was silenced in these cells (Figure 3A, lane 4 in CL1–5 and CL141). Silencing MDA-9/Syntenin expression down-regulated the activities of endogenous Slug and increased E-cadherin expression (Figure 3A, lane 2 in CL1–5 and CL141). In accordance with this observation, silencing MDA-9/Syntenin expression in CL1–5 and CL141 inhibited cancer cell invasiveness in both parental and Slug-overexpressing cells (Figure 3B).

To further demonstrate whether the expression of MDA-9/Syntenin was required for Slug-mediated cancer metastasis *in vivo*, CL1–5/vector and CL1–5/Slug stable cells with or without MDA-9/Syntenin silencing were established by viral infection, and cell invasion capacity was confirmed (Supplementary Figure 2B). Four groups of tumor cells were inoculated directly into the tail vein of mice and the formation of pulmonary nodules in mice was examined at 35 days. Mice with CL1–5/Slug+shCtl cells developed more pulmonary nodules than those injected with CL1–5/vector+shCtl cells (mean number: 56.33 ± 47.87 for line vector+shCtl and129.66 ± 45.45 for line Slug+shCtl; *p* < 0.05) (Figure 3C–3D). In contrast, mice injected intravenously with MDA-9/Syntenin silencing clones developed fewer pulmonary nodules (mean number: 34 ± 12.47 for line Vector+shSyntenin-b and 78.33 ± 29.25 for line Slug+shSyntenin-b, *p* < 0.05) (Figure 3D). Thus, pulmonary metastasis of the CL1–5 murine model supported the findings that MDA-9/Syntenin expression regulated Slug-mediated cancer metastasis *in vivo*.

### Slug interacts with the PDZ1 domain of MDA-9/Syntenin by linker region

**Figure 4:**
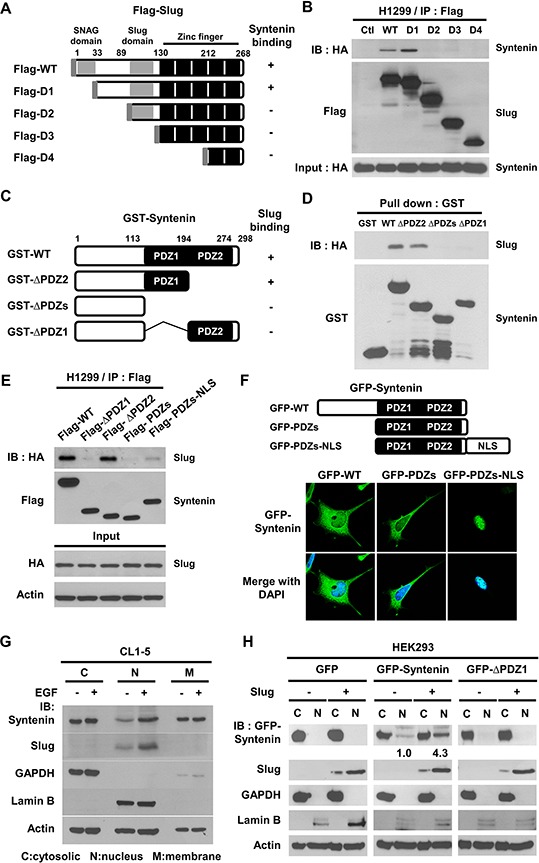
The PDZ1 domain and nucleus localization of MDA-9/Syntenin were required to interact with Slug **A.** Schematic diagram showing the structure of wild-type Slug and its deletion constructs. **B.** After transient transfection with truncated Slug variants with HA-Syntenin in H1299 cells, the lysates were immunoprecipitated with anti-Flag antibodies and resolved by SDS-PAGE. **C.** Schematic graph representing the domain structure of MDA-9/Syntenin and its three deletion mutants. **D.** The indicated purified GST-Syntenin fusion proteins were incubated with *in vitro* translated proteins of HA-tagged Slug and pulled down by glutathione beads. The protein association was determined by immunoblotting. **E.** After transient transfection with variants of Flag-Syntenin and HA-Slug in H1299 cells, the lysates were immunoprecipitated with anti-Flag antibodies and resolved by Western blotting. **F.** The confocal images of HEK293 cells transiently transfected with pEGFP-Syntenin, pEGFP-PDZs, and pEGFP-PDZs-NLS plasmids, respectively. **G.** CL1–5 cells were cultured in serum-free medium for 12 h with or without EGF (40 ng/ml) treatment for 6 h. The lysates from cell fractionation were resolved by immunoblotting. **H.** Cell fractionation analysis of HEK293/vector or HEK293/Slug cells transiently transfected with EGFP, EGFP-Syntenin, and EGFP-ΔPDZ1 plasmids.

To examine whether MDA-9/Syntenin enhanced EMT by association with Slug, a series of deletion mutants were generated to determine the reciprocal interacting domains between Slug and MDA-9/Syntenin (Figure 4A). The linker region of Slug around residue amino acid 33–89, between the SNAG and the Slug domain, was required for the interaction with MDA-9/Syntenin (Figure 4B). On the other hand, results of *in vitro* GST pull-down assays by different deletion constructs of MDA-9/Syntenin (Figure 4C and Supplementary Figure 3A) showed that Slug proteins were only present in the pull-down complexes of PDZ1-containing MDA-9/Syntenin (Figure 4D and Supplementary Figure 3A). This observation was consistent with the yeast-two hybrid screening in which a fragment of MDA-9/Syntenin encompassing the PDZ1 domain was associated with Slug (Supplementary Figure 1A).

To further study if the PDZ1 domain of MDA-9/Syntenin was essential for the association with Slug, immunoprecipitation results showed that Flag-tagged WT- and ΔPDZ2-Syntenin were associated with Slug (Figure 4E and Supplementary Figure 3B), but the PDZ1-containing mutant, Flag-PDZs, with lower nucleus localization property [29], was not. When Flag-PDZs expression was forced into the nucleus by tagging a nuclear-leading sequence (NLS) to the C-terminus of PDZs (Figure 4F), there was increased association with Slug (Figure 4E lane 5), implying that the nuclear localization of MDA-9/Syntenin was crucial for binding to Slug.

### Mitogen EGF stimulation increases MDA-9/Syntenin nuclear translocation

To explore whether mitogen stimulation regulated nuclear translocation of MDA-9/Syntenin in lung cancer cells, CL1–5 cells were treated with recombinant epidermal growth factor (EGF) and the distribution of MDA-9/Syntenin was analyzed. Treatment of EGF elevated Slug protein expression and markedly increased MDA-9/Syntenin level in the nuclear fraction (Figure 4G and Supplementary Figure 4B).

To examine whether Slug expression affected the distribution of MDA-9/Syntenin, GFP-tagged Syntenin deletion constructs were co-expressed with DsRED-tagged Slug fusion proteins in HEK293 cells. More Syntenin-WT and -ΔPDZ2 accumulated in the nucleus of Slug-expressing cells than Syntenin-ΔPDZ1 and -PDZs (Supplementary Figure 4A). To confirm these observations, Syntenin-WT and -ΔPDZ1 were transfected in the control or Slug-overexpressing stable cells. The distribution of MDA-9/Syntenin proteins was detected by cell fractionation. The control cells showed that the distribution of Slug was largely detected in the nuclear fraction and GFP proteins in the cytosolic fraction (Figure 4H). The level of nuclear GFP-Syntenin in Slug-overexpressing stable cells increased 4.3-fold compared to that of the control cells. This increase was abolished by Syntenin-ΔPDZ1 expression (Figure 4H). Thus, the association with Slug facilitated MDA-9/Syntenin maintenance in the nucleus.

### MDA-9/Syntenin enhances Slug-mediated transcription repression through HDAC1 recruitment

Since MDA-9/Syntenin binds to Slug, how MDA-9/Syntenin regulated Slug function was further addressed. It had been reported that MDA-9/Syntenin could enhance the transcription activity of Sox4 by decreasing its proteosomal degradation [31, 32]. Thus, the half-life of Slug protein in the control or MDA-9/Syntenin-expressed cells was estimated by cycloheximide assay. The results showed that the stability of Slug protein was not significantly altered (Supplementary Figure 5A).

**Figure 5:**
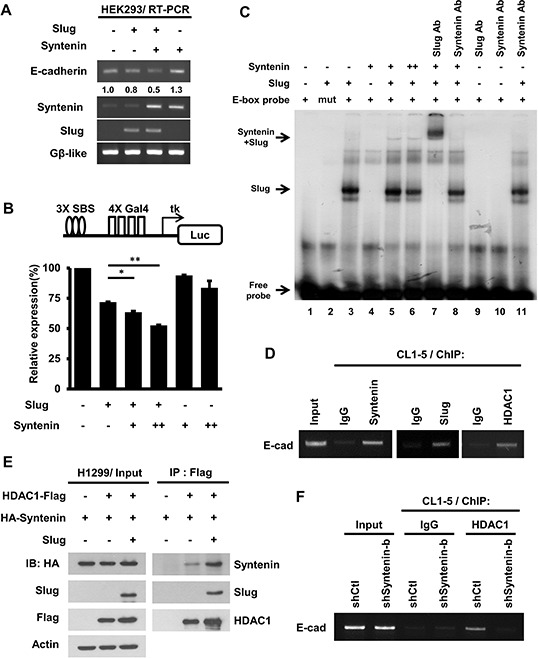
MDA-9/Syntenin facilitated Slug-dependent transcription repression by recruiting the co-repressor, HDAC1 **A.** The E-cadherin mRNA level was determined by RT-PCR in HEK293 cells expressing control, Slug, MDA-9/Syntenin, or Slug+MDA-9/Syntenin. The expression level of Gβ-like was used as the internal control. **B.** HEK293 cells were transfected with 3xSBS luciferase reporter with plasmids expressing Slug and Flag-Syntenin for 48 h. Bar graph represents the relative expression of luciferase activity normalized to the control renilla reporter activity ± SD (*n* = 3, *p < 0.05, **p < 0.001). **C.** MDA-9/Syntenin was associated with Slug on the E-box oligonucleotides. Phospho-image analysis of the EMSA gel showing ^32^P-labeled E-box oligonucleotides incubated with *in vitro* translation proteins (+: 3 μl, ++: 6 μl) or with the indicated antibodies (Ab: antibody, 0.3 μg). **D.** ChIP-PCR analyses of MDA-9/Syntenin, Slug, and HDAC1 on the E-cadherin promoter in CL1–5 cells. Endogenous MDA-9/Syntenin, Slug, and HDAC1 were detected by ChIP with the indicated antibodies. **E.** At 48 h post-transfection of the plasmids expressing the indicated HDAC1-Flag, HA-Syntenin, Slug proteins in H1299 cells, and the cell lysates were immunoprecipitated with anti-Flag antibodies. The associated proteins were then analyzed by Western blotting. **F.** The CL1–5/shCtl and CL1–5/shSyntenin-b stable cells were examined for HDAC1 level on the E-cadherin promoter. ChIP assays were performed using anti-HDAC1 antibodies, with IgG as negative control.

However, the mRNA of E-cadherin was reduced in Slug-expressing cells when co-expressed with MDA-9/Syntenin (Figure 5A). To clarify whether MDA-9/Syntenin enhances Slug repressor activity, Slug with or without MDA-9/Syntenin was transfected in a 3x-snail binding site (SBS)-Gal4 luciferase reporter system in HEK293 cells. Slug repression activity was significantly increased by 36.5% and 47.4%, respectively, when MDA-9/Syntenin expressions were dose-dependently elevated (Figure 5B).

To explore whether MDA-9/Syntenin affects Slug binding to the E-box sequence, EMSA was used. HA-Slug, but not HA-Syntenin, directly bound to the E-box sequence (Figure 5C, lane 3 and 4)). However, HA-Syntenin was associated with HA-Slug on the E-box probe (Figure 5C, lane 5). The results suggest that MDA-9/Syntenin is involved in the Slug repression complex by binding to the promoter region of Slug's target indirectly.

To determine if MDA-9/Syntenin modulated Slug-mediated E-cadherin transcriptional repression, ChIP assay was performed in CL1–5 cells. MDA-9/Syntenin, Slug, and HDAC1 were involved in the E-cadherin promoter region *in vivo* (Figure 5D). Furthermore, the association of MDA-9/Syntenin with HDAC1 was observed *in vitro* and *in vivo* (Supplementary Figure 5B and Figure 5E). Moreover, HDAC1 on the E-cadherin promoter was reduced in MDA-9/Syntenin-knockdown cells, implying that MDA-9/Syntenin acted as a mediator to facilitate the binding of HDAC1 on the E-cadherin promoter (Figure 5F). These data suggest that MDA-9/Syntenin enhances the repression activity of Slug by recruiting the co-repressor, HDAC1, to the E-box element.

### MDA-9/Syntenin-enhanced Slug-mediated E-cadherin suppression and cancer invasion is dependent on its nuclear localization and co-repressor recruitment

**Figure 6:**
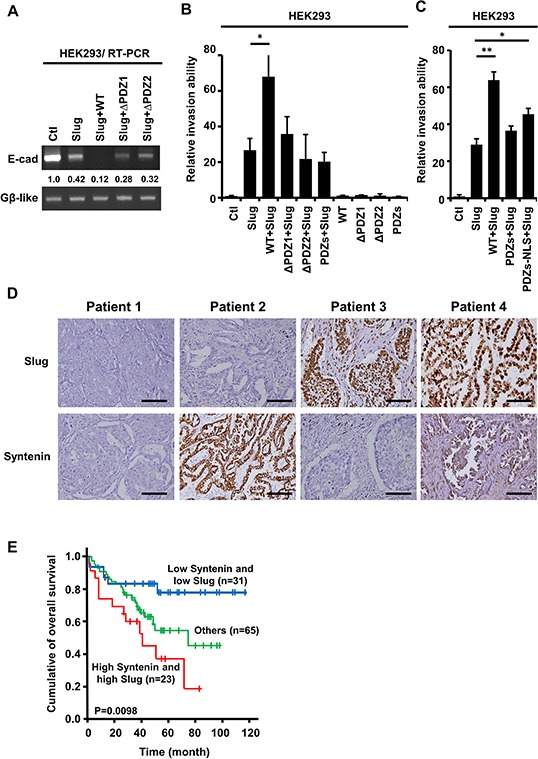
The PDZ domains of nuclear MDA-9/Syntenin were required to enhance Slug-regulated cell invasion and the expressions of Slug/Syntenin were associated with poor survival in patients with lung adenocarcinoma **A.** E-cadherin mRNA level was determined by RT-PCR in HEK293 cells expressing control, Slug, Slug+WT, Slug+ ΔPDZ1, or Slug+ΔPDZ2. **B.** HEK293 cells were infected with the indicated MDA-9/Syntenin deletion mutants with control vector or Slug for 72 h. The invasive capacity of these cells was determined using the modified Boyden chambers invasion assay. Invasive cells are represented as mean ±SD (*n* = 3, *p < 0.05). **C.** The HEK293/vector or HEK293/Slug cells were transiently transfected with Flag-WT, Flag-PDZs, and Flag-PDZs-NLS of MDA-9/Syntenin for 48 h. The invasive cells of these transfected cells were scored by the modified Boyden chambers invasion assay. The bar graph shows the mean number of invasive cells ± SD (*n* = 3, *p < 0.05, **p < 0.001). **D.** Immunohistochemical analysis of typical protein expression patterns of Slug and MDA-9/Syntenin in primary tumor specimens from 119 patients with lung adenocarcinoma who underwent surgery. Scale bars, 100 μm. **E.** Kaplan-Meier analysis of the overall survival for the 119 NSCLC patients with Slug^−^Syntenin^−^, Slug^+^Syntenin^−^, Slug^−^Syntenin^+^, and Slug^+^Syntenin^+^. The *p* values were performed by two-sided log-rank tests.

The PDZ domains of MDA-9/Syntenin were required for Slug-mediated E-cadherin suppression (Figure 6A). To determine whether the enhanced Slug-mediated cell invasion requires PDZ domains and the nuclear presence of MDA-9/Syntenin, Slug-mediated cell invasiveness was detected in the presence of each MDA-9/Syntenin deletion mutant in HEK293 cells, and the protein expression levels was shown in Supplementary Figure 6A. The result showed that Syntenin-ΔPDZ1, which abolished the interaction with Slug, resulted in a complete loss of enhanced Slug-mediated cell invasion (Figure 6B). Noticeably, Syntenin-PDZs abrogated invasion, whereas Syntenin-PDZs-NLS, which showed mild association with Slug, partially enhanced invasion (Figure 6C), implying that nuclear localization of MDA-9/Syntenin is crucial in enhancing Slug-mediated cancer invasion. Furthermore, Syntenin-ΔPDZ2 did not increase cell invasiveness and enhanced Slug-mediated E-cadherin suppression even though this mutant was translocalized at the nucleus (Supplementary Figure 4A) and bind to Slug (Figure 4E). These findings suggest that both the PDZ domains of MDA-9/Syntenin and nuclear localization are needed to promote Slug-mediated cell invasion. MDA-9/Syntenin can act as a pivotal adaptor to enhance Slug-mediated cell invasion (Supplementary Table 1).

### High MDA-9/Syntenin and high Slug expressions are associated with poor survival in patients with lung adenocarcinoma

To further verify the clinical significance of MDA-9/Syntenin and Slug expression in cancer progression, both MDA-9/Syntenin and Slug protein expressions were examined by immunohistochemistry in tumor specimens obtained from 119 patients with lung adenocarcinoma. The characteristics of the 119 patients were provided in Table 1. Serial sections of each specimen were stained with antibodies against MDA-9/Syntenin and Slug (Figure 6D). Analysis of the combined effects of both MDA-9/Syntenin and Slug proteins on patients' prognoses revealed that patients with high MDA-9/Syntenin and Slug expressions had poorer overall survival than those with low expressions (Figure 6E, *p* = 0.0098).

**Table 1:** MDA-9/Syntenin and Slug expression in relation to clinical parameters and pathological characteristics^1^

			Slug			Syntenin		
Category	Subcategory	Number	Low (%)	High (%)	*P*	Low (%)	High (%)	*P*
Total patients		119	65(54.6)	54(45.4)		62(52.1)	57(47.9)	
Sex	Female	55	32(49.2)	23(42.6)	0.470	26(41.9)	29(50.9)	0.328
	Male	64	33(50.8)	31(57.4)		36(58.1)	28(49.1)	
Tumor size, cm	< 3	24	13(20.0)	11(20.4)	0.960	13(21.0)	11(19.3)	0.821
	≥ 3	95	52(80.0)	43(79.6)		49(79.0)	46(80.7)	
Tumor stage	Stage I	54	35(53.8)	19(35.2)	0.005	22(35.5)	32(56.1)	0.068
	Stage II	39	13(20.0)	26(48.1)		23(37.1)	16(28.1)	
	Stage III	26	17(26.2)	9(16.7)		17(27.4)	9(15.8)	
Slug expression (%)	Low	65	-	-		31(50.0)	34(59.6)	0.291
	High	54	-	-		31(50.0)	23(40.4)	
Syntenin expression (%)	Low	62	31(47.7)	31(57.4)	0.291	-	-	
	High	57	34(52.3)	23(42.6)		-	-	

1 MDA-9/Syntenin and Slug expression were designated as ‘high’ or ‘low’ using 10% immunoreactivity in tumor sections as cut-off point (low MDA-9/Syntenin and low Slug as the reference), and these values were adjusted according to sex, tumor size and tumor stage. The *P* values were calculated using Pearson's chi-square test.

Multivariable Cox proportional-hazards regression analyses with a stepwise selection model were made to evaluate the associations of various independent prognostic factors with patient survival (Table 2). The phenotype of both high MDA-9/Syntenin and Slug expressions was an independent predictor (hazard ratio: 2.348, 95%CI: 1.412–3.906; *p* = 0.001) after controlling for all other prognostic factors. These results suggest that patients with both high MDA-9/Syntenin and high Slug expressions is associated with worse clinical outcomes in lung adenocarcinoma.

**Table 2:** Hazard ratios for mortality in patients with lung adenocarcinoma, determined by immunohistochemistry staining according to multivariable Cox regression analysis^1^

Variable	Hazard ratio (95% C.I.)	*P*
MDA-9/Syntenin+Slug	2.348 (1.412 to 3.906)	0.0010
Age	0.978 (0.948 to 1.009)	0.1557
Sex	3.012 (1.536 to 5.907)	0.0013
Tumor stage	1.431 (0.656 to 3.124)	0.3681

1 MDA-9/Syntenin and Slug expressions were designated as ‘high’ or ‘low’ using 10% immunoreactivity in tumor sections as the cut-off point. These values were adjusted according to age, sex, and tumor stage. The *p* value was calculated using Pearson's chi-square test.

Abbreviation: CI, confidence interval

## DISCUSSION

**Figure 7:**
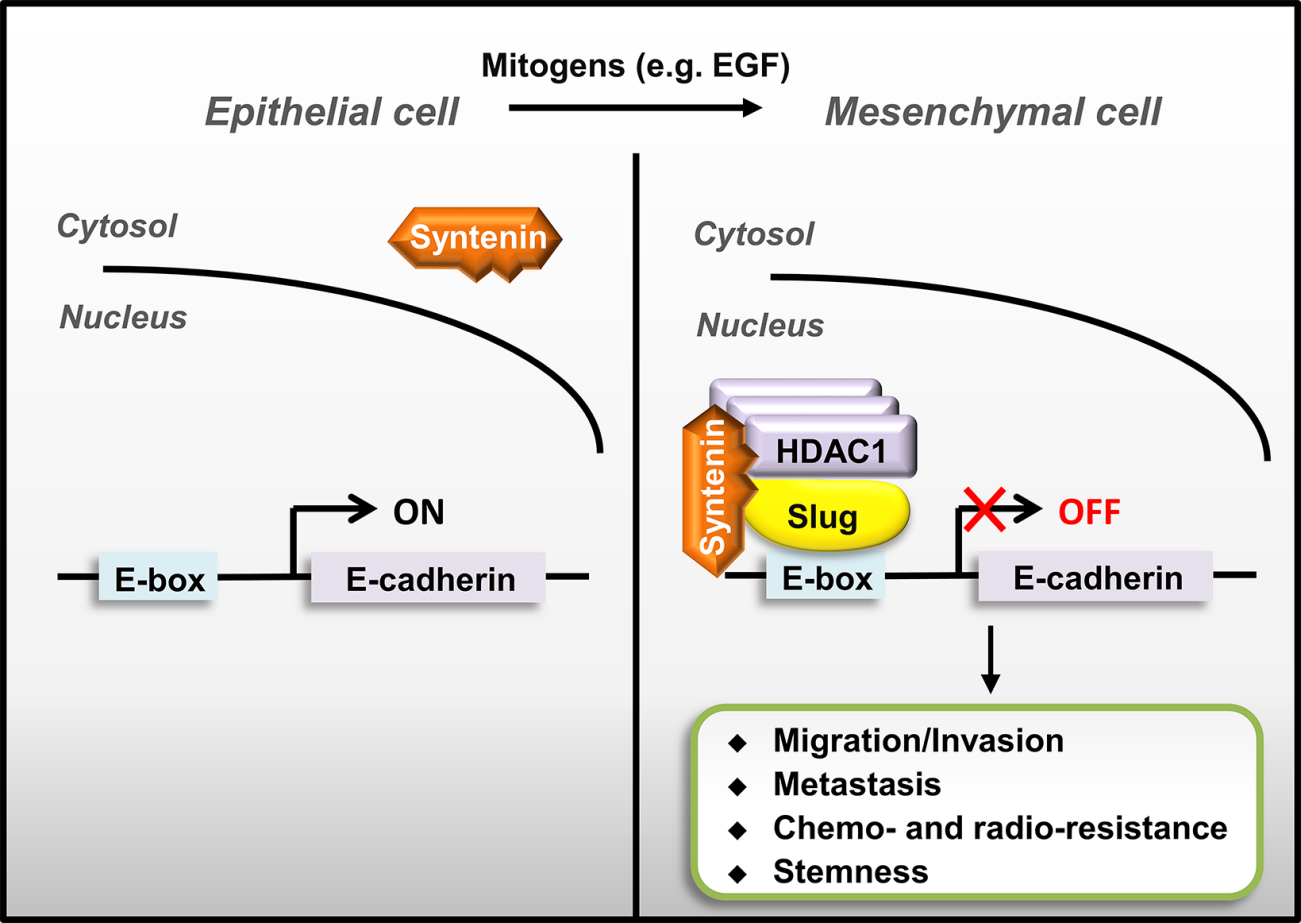
A schematic model of nuclear MDA-9/Syntenin enhances Slug-mediated cancer cell invasion through transcription co-repressor recruitment Aberrant Slug expression accumulated MDA-9/Syntenin in the cell nucleus by binding to MDA-9/Syntenin and then recruiting co-repressors to repress adhesion molecules, such as E-cadherin, thereby enhancing Slug-mediated EMT/invasion to promote cancer metastasis.

MDA-9/Syntenin has recently been identified to play an important role in controlling cancer invasion and progression [19]. Aberrant expressions of MDA-9/Syntenin are associated with poor clinical outcome in glioma, breast cancer, and uveal melanoma [22, 23, 30]. MDA-9/Syntenin is also involved in the biogenesis of exosome and modulation of cancer microenvironment [18, 33, 34], suggesting that it may be a promising therapeutic target [35]. Our study identified that nuclear MDA-9/Syntenin can act as a co-repressor in Slug-mediated EMT in lung adenocarcinoma (Figure 7). MDA-9/Syntenin elevates its nuclear location during mitogen-mediated signaling activation and enhances the co-repressor recruitment to the Slug transcription repression complex, thereby modulating Slug-mediated cancer progression.

The nuclear translocation of MDA-9/Syntenin is a prerequisite to exert its enhancement of Slug repressor functions. Previous studies and our finding indicate that EGF stimulates Slug activity by protein stabilization [11], and then promotes MDA-9/Syntenin accumulation in the cell nucleus (Figure 4G and 4H). The increased nuclear MDA-9/Syntenin can then interact with Slug through the PDZ1-interacting domain and can enhance Slug transcriptional activity. However, Slug over-expression retains MDA-9/Syntenin in the nucleus, like other MDA-9/Syntenin-binding transcription factors, by an unknown mechanism [31].

Moreover, MDA-9/Syntenin augments Slug transcriptional repression activity by associating with the co-repressor, HDAC1. The Syntenin-ΔPDZ2 presumably lacks the ability to recruit HDAC1, and thus is unable to increase cell invasion. Taken together, these results indicate that this regulation is dependent on nuclear translocation and co-repressor recruitment by MDA-9/Syntenin.

Nuclear MDA-9/Syntenin distribution was first reported by Pascale *et al.* [29]. Additional studies have pointed out that nuclear MDA-9/Syntenin can be detected in highly metastatic cell lines and tumor specimens [29, 30, 36], but the detailed mechanism of how nuclear MDA-9/Syntenin is involved in cancer metastasis remains unclear. The present study demonstrates a potential mechanism by which nuclear MDA-9/Syntenin may accumulate in the cell nucleus by binding to a transcription repressor and recruiting more cofactors to enhance their biological function in cells. Since there is no typical nuclear localization signal in the sequence of MDA-9/Syntenin, how MDA-9/Syntenin is translocated into the cell nucleus should be further clarified.

Although the expression of MDA-9/Syntenin is ubiquitous, the associations with other proteins can affect the distribution of MDA-9/Syntenin [37]. The two PDZ domains of MDA-9/Syntenin contain distinct peptide-binding specificities and the C-terminal region may have membrane targeting regulation capability [19, 29, 38]. The N-terminal region of MDA-9/Syntenin can bind to Sox4 or ubiquitin and facilitate MDA-9/Syntenin localization in either the cytoplasm or nucleus [31, 36]. This study shows that loss of the N-terminal region of MDA-9/Syntenin diminishes its nucleus accumulation and protein association with Slug (Supplementary Figure 4a, 3a, and Figure 4e), and reduces the capability to enhance Slug-mediated cell invasion. The intra-molecular interaction of the unstructured N-terminal region of MDA-9/Syntenin affects ligand binding to the PDZ domains and restricts the membrane targeting ability of MDA-9/Syntenin. These findings suggest that the N-terminal extension of MDA-9/Syntenin may have more functions in regulating sub-cellular targeting.

Slug has been reported as a critical promoter of cancer progression, however, the regulation mechanism of Slug-mediated transrepression remains unclear. Previous studies indicate that HADC1 is required for Slug-mediated gene silencing [39], and our finding provides a new insight into Slug-mediated EMT regulation in which MDA-9/Syntenin facilitates the recruitment of HDAC1 to enhance the repression activity of Slug, thereby results in increased cell invasiveness and metastasis *in vivo*. Furthermore, the expression levels of both MDA-9/Syntenin and Slug can predict clinical outcome in lung adenocarcinoma patients. Thus, the MDA-9/Syntenin/Slug regulatory pathway may serve as an important therapeutic target for lung adenocarcinoma.

## MATERIALS AND METHODS

### Cell culture

The human lung adenocarcinoma cell lines A549, H1299, and the embryonic kidney cell lines HEK293, and HEK293T cells were purchased from the American Type Culture Collection (ATCC, Manassas, Virginia). The human lung adenocarcinoma cell lines EKVX, H322M, H522, H226, H520, HOP62, and H23, and breast cancer cell lines MCF-7, T47-D, MDA-MB-231, and Hs578t were purchased from the Developmental Therapeutics Program of the National Cancer Institute (NCI, Bethesda, Maryland).

The human lung adenocarcinoma cell lines CL1–5 and CL141 were established in the study laboratory, CL1–5 derived from CL1–0 cell line by *in vitro* transwell selection as previously described [40] and CL141 was derived from clinical patients [41]. The cell lines H1299, A549, MCF-7, Hs578t, HEK293, and HEK293T were cultured in DMEM supplemented with 10% FBS at 37°C in a humidified incubator containing 5% CO2. The EKVX, H23, H322M, H522, H226, H520, HOP62, CL1–5, CL141, T47-D, and MDA-MB-231 were cultured in RPMI 1640 medium supplemented with 10% FBS.

### Plasmids and antibodies

Human MDA-9/Syntenin cDNA were obtained from H1299 by RT-PCR. Its full length or deletion constructs were sub-cloned into pACT2, pcDNA3.1-HA, pCMV-tag2 (Clontech, CA, USA), pEGFP (Invitrogen), and pLKO.1-AS2-neo vectors for the production of lentivirus or pET28a vectors for bacterial expression by standard molecular cloning procedure. Flag-tagged Slug was as described previously [10]. Full length or deletion constructs of Slug were sub-cloned into pAS2–1, pCI-neo, pcDNA3.1-HA, pDsRED, and AS2-neo vectors. pcDNA3-HDAC1-Flag was kindly provided by Dr. Wen-Ming Yang (Institute of Molecular Biology, National Chung Hsing University, Taichung, Taiwan).

The primary antibodies used for immunoblot analysis were anti-Syntenin (mouse monoclonal, S-31 or rabbit polyclonal, H-48, Santa Cruz Biotechnology, CA, USA and rabbit monoclonal, Abcam, MA, USA), goat anti-Slug (Santa Cruz Biotechnology), goat anti-HDAC1 (Santa Cruz Biotechnology) and mouse anti-β-actin (Santa Cruz Biotechnology), mouse anti-Flag (Sigma-Aldrich, MO, USA), mouse anti-HA (Covance, Berkeley, California, USA), mouse anti-E-cadherin (BD Biosciences, Inc., CA, USA), mouse anti-vimentin (BD Biosciences), mouse anti-N-cadherin (BD Biosciences), and mouse anti-GFP (BD Biosciences).

### Experimental metastasis *in vivo*

The single-cell suspension of CL1–5/vector or CL1–5/Slug with MDA-9/Syntenin-silenced expression cells (1 × 10^6^) containing in 0.1 ml of PBS was injected into the lateral tail veins of 6-week-old NOD SCID mice (Bio Lasco, Taiwan Co., Ltd., Taipei, Taiwan). After 35 days, the four groups of tumor cell-injected mice were sacrificed and their lungs examined for metastasis (*n* = 6 per group). The lungs were removed and fixed in 10% formalin. The number of lung nodules were counted using a dissecting microscope.

### Chromatin immunoprecipitation (ChIP)

CL1–5, CL1–5/shCtl, or CL1–5/shSyntenin-b (1 × 10^7^) cells were analyzed by Magna ChIP™ A/G (Millipore, Billerica, MA) according to the manufacturer's instructions. Briefly, the cells was cross-linked in culture media with 1% formaldehyde and quenched by 0.125 M glycine. After washing with cold PBS, the cells were scraped and soluble chromatin lysates were extracted by sonication and centrifugation, and then mixed with anti-Slug (Santa Cruz), anti-HDAC1 (Millipore), or anti-Syntenin (Santa Cruz) antibodies and protein A/G magnetic beads overnight at 4°C. The complexes were pelleted and washed with indicated buffers.

The DNA/protein solution was eluted with proteinase K containing elution buffer at 65°C for 2 h to reverse the formaldehyde cross-links. The DNA elutes were purified and E-cadherin-specific amplified by PCR using indicated primers (forward, 5′-CGAACCCAGTGGAATCAGAA-′3 and reverse, 5′-GCGGGCTGGAGTCTGAACTG-3′).

### Patients and tumor specimens

The Institutional Review Board of National Taiwan University Hospital (NTUH, Taipei, Taiwan) approved this study and all of the participants provided written informed consent. Lung tumor tissue specimens were obtained from patients (*n* = 119) with histologically-confirmed lung adenocarcinoma who had undergone complete surgical resections at the NTUH between 1995 and 2005. All of the specimens were formalin fixed, sectioned, stained with hematoxylin and eosin, and examined by microscopy. Pathologic staging was performed by a single pathologist according to the international staging system for lung cancer.

### Immunohistochemistry

Immunohistochemical staining of tumor tissue samples from patients with adenocarcinoma was conducted as previously described. Briefly, sections for analysis of Slug or MDA-9/Syntenin protein expressions were first autoclaved in Trilogy Solution (Cell Marque Corp) or Antigen Retrieval Citra Solution (Biogenex, San Ramon, CA) at 121°C for 10 min. The samples were subsequently treated with 3% H_2_O_2_-methanol and incubated with DakoCytomation Dual Endogenous Enzyme Block (DakoCytomation) for 10 min, Ultra V Block (Lab Vision Corporation) for 10 min, antibody-dilution buffer (Ventana Medical Systems, Inc., Tucson, AZ) for 10 min, and with anti-Slug (1:75, ABGENT) antibody at room temperature or the anti-MDA-9/Syntenin antibody (1:100, Santa Cruz Biotechnology) overnight at 4°C.

Immunostaining was detected using a Super Sensitive Non-Biotin Polymer HRP Detection System (BioGenex, San Ramon, CA) according to the manufacturer's protocol.

### Statistical analysis

Data were shown as mean ± SEM. Statistical analyses were performed by the Student's *t*-test or Pearson's χ2 test, as appropriate. Overall survival for patient groups with different expression signatures was determined using the Kaplan-Meier method and two-sided log-rank test. Immunoreactivity in more than 10% of the tumor specimens was defined as high-level Slug and MDA-9/Syntenin expression. Statistical significance was set at *p* < 0.05. All statistical analyses were performed using the SPSS software (v10.0; SPSS, Inc., Chicago, IL).

## SUPPLEMENTARY DATA FIGURES AND TABLES


