# Supplementary material for: MDA-9/Syntenin-Slug transcriptional complex promote epithelial-mesenchymal transition and invasion/metastasis in lung adenocarcinoma
Source: Oncotarget. 2015 Nov 2;7(1):386–401. doi: 10.18632/oncotarget.6299 (PMC4808006; doi:10.18632/oncotarget.6299)
Supplement: Supplementary file 1 [file oncotarget-07-0386-s001.pdf]

SUPPLEMENTARY DATA

EXPERIMENTAL PROCEDURES

Yeast two-hybrid

Yeast two-hybrid screening was performed as previously described [1]. Yeast strain L40 cells transformed with pBTM116-Slug were mated with yeast cells, which were transformed with prey genes from human prostate cancer cDNA library in the pACT2 vector, the kindly gift from Dr. Hsiu-Ming Shih (Institute of Biomedical Sciences, Academia Sinica, Taipei, Taiwan). The resulting mated yeast cells were selected on a medium lacking histidine, leucine, and tryptophan in 384-well plates. Yeast strain AH109 cells were co-transformed with pACT2-Syntenin and pAS2–1-Slug or pAS2-α-catulin and the transformed yeast cells were grown on a yeast drop-out media lacking the indicated selection medium. The samples were incubated at 30°C for 5–7 days.

Transfection and viral infection

Transfection of plasmid DNA was performed using Lipofectamine or Lipofectamine 2000 (Invitrogen, Carlsbad, CA, USA) according to the manufacturer's instructions. For transient knockdown experiments, the CL1–5 or CL141 cells were conducted with the siRNA targeting of human MDA-9/Syntenin, Slug, and low GC-matched negative universal control # 3 (Invitrogen) by RNAiFect® transfection reagent (Qiagen,Valencia, CA) according to manufacturer's protocols. The sequences of the indicated siRNA designation were listed in Supplementary Table 1. The cells were used for all experiments 48 hours post-transfection.

Lentiviral vector expressing siRNA against human MDA-9/Syntenin (shSyntenin-a and shSyntenin-b), Slug (shSlug) and its control (shLacZ) were obtained from the RNAi consortium at Academia Sinica. The target sequence of each shRNA to human gene was listed in Supplementary Table 2. For lentiviral production, HEK293T cells were co-transfected with the indicated lentiviral vector [Flag-tagged, untagged Syntenin (WT, ΔPDZ1, ΔPDZ2, PDZs, and PDZS-NLS), Slug, shSyntenin, shSlug and shLacZ] with two helper plasmids (pMD.G and pCMVΔ8.91) using Lipofectamine 2000 reagents according to the manufacturer's protocols.

Virus-containing medium was collected at 24, 48, and 72 h post-transfection, centrifuged, and filtered through 0.45 mm-pore-size filters. HEK293, CL1–5 and CL141 cells were infected with the indicated lentivirus in media containing polybrene (8 μg/ml). At 24 h post-infection, the cells were replaced with fresh medium for 48 h and then used for all of the experiments.

Immunoprecipitation and immunoblotting

For co-immunoprecipitation, H1299 cells were transfected with the indicated plasmids for 48 h. For endogenous co-immunoprecipitation assays, CL1–5 cells were cross-linked with 3 mM Dimethyl 3,3′-dithiobisproprionamidate-2-HCl (DTBP, Pierce, IL). The cells were lysed in IP lysis buffer (20 mM Tris [pH 7.5], 100 mM NaCl, 1% NP-40, 100 μM Na3VO4, 50 mM NaF, and 30 mM sodium pyrophosphate) containing protease inhibitors with or without EDTA (Roche Diagnostics, Mannheim, Germany). An aliquot of cell lysate was incubated with the indicated antibodies at 4°C overnight, and then with protein A-Sepharose or protein G beads (Sigma-Aldrich, MO, USA) for 1 h at 4°C.

The beads were then collected and washed three times in IP lysis buffer and eluted by boiling in SDS sample buffer. The protein elutes were separated by SDS-PAGE and processed for immunoblotting using the relevant primary antibodies.

Modified boyden chamber invasion assay

Modified Boyden chambers with polycarbonate-membrane inserts (pore size 8 mm; BD Falcon, Franklin Lakes, NJ) and coated with Matrigel (BD Biosciences) (12 μg for HEK293 and 30 μg for CL1–5 and CL141) were performed for cell invasion assays. Cells (2.5×10^4) suspended in DMEM or RPMI medium containing 10% Nu-Serum (Invitrogen) were plated in the upper chambers. After 24 h incubation at 37°C, the cells were fixed with methanol and stained with a 50 μg/ml solution of propidium iodide (Sigma-Aldrich, MO, USA) at room temperature for 30 min. Each membrane was photographed and the number of cells counted under a microscope at 650 magnification using the Analytical Imaging Station software package (Imaging Research Inc, St Catherine's, Canada). Each experiment was assayed in triplicate.

Cell fractionation

The MDA-9/Syntenin and Slug distribution in cells were analyzed by Qproteome Cell Compartment Kit (Qiagen) according to the manufacturer's protocol. The cells were scraped and washed by cold PBS twice. The cytosolic, membrane, and nuclear fractions were obtained from centrifuged cell pellets incubated with the extraction buffers at 4°C. The proteins of the cell fraction were detected by SDS-PAGE and by immunoblotting.

Reverse-transcriptase PCR

The total RNA of cells was extracted by Trizol (Invitrogen) and cDNAs were prepared and underwent PCR as described previously. The primer sequences used were listed in Supplementary Table 3. The PCR product of each sample was determined by agarose gel electrophoresis.

Expression of GST fusion proteins and GST pull-down assay

The recombinant GST-Syntenin and its deletion derivatives were induced in XA-90 bacterial strain with 0.5 mM IPTG for 30 minutes, and then purified with glutathione beads (GE Healthcare/Amersham Biosciences, Buckinghamshire, UK) according to standard protocol. HA-Slug and HDAC1-Flag were produced by TNT® Quick Coupled Transcription/Translation Systems (Promega, Madison, WI). HA-Slug or HDAC1-Flag was mixed with purified GST-Syntenin or its variants. Pull-down assays were performed at 4°C for 1 h. The beads were then washed thoroughly with PBS buffer. The bound proteins were eluted by boiling in SDS sample buffer, separated by SDS-PAGE, and detected by immunoblotting.

Luciferase reporter assay

The H1299 cells (8×10^4) were co-transfected with 100ng 3xSBS-luciferase reporter plasmid, 250 ng pGal4-VP16, 50ng pCI-neo-Slug, pCMV-Tag2-Syntenin (0.5 µg or 1 µg), and 50 ng pGL4-TK-renilla for normalization of transfection efficiency. After transfection for 36 h, the cell lysates were collected and the activity of firefly luciferase and renilla luciferase were measured by a dual-Luciferase reporter assay system (Promega, Madison, WI).

Immunofluorescence

The transfected cells that expressed EGFP-Syntenin and DsRed-Slug were fixed with methanol/acetone (1:1) for 5 min and washed with PBS. All of the samples were mounted onto microscope slides with ProLong Gold anti-fade reagent with DAPI (Invitrogen Life Technologies) and examined and photographed by an LSM 510 laser scanning confocal microscope (Carl Zeiss).

Electrophoretic mobility shift assay (EMSA)

To radiolabel oligonucleotide targets, 1.25 pmol annealed oligonucleotide (IDT DNA) were labeled at the 5′ end with ATP (Amersham, Buckinghamshire, UK) by T4 polynucleotide kinase according to the manufacturer's protocol (Life Technologies). Free nucleotides were removed using ProbeQuant G-50 micro columns (GE Healthcare). The duplex oligonucleotide sequences used were E-BoxC, 5-GACTTCCGCAAGCTCACAGGTGCTTTGCAGTT CCGACG-3 and mutant E-BoxC, 5-GACTTCCGCAAGCTCATAGGTTCTTTGCAGTTC CGACG-3. The HA-Slug and HA-Syntenin proteins for EMSA were prepared by TnT® Quick Coupled Transcription/Translation System (Promega).

In EMSA, the synthesized proteins (2 µl HA-Slug with 2.5 or 5 µl HA-Syntenin) were combined with 25 g/mL poly-dI/dC (Sigma-Aldrich), 5 µl EMSA binding buffer (20 mM Hepes, pH 7.6, 150 m M KCl, 3 mM MgCl2, 0.5 mg/mL BSA, 10% glycerol, and 0.2 mM ZnSO4), and anti-Syntenin or anti-Slug antibody at room temperature for 15 min. After incubation, 1 µl radio-labeled oligonucleotide probe was added and the proteins were incubated at room temperature for another 30 min. Protein-DNA complexes were resolved on a 6%TBE/acrylamide gel.

REFERENCES

1. Liang CH, Chiu SY, Hsu IL, Wu YY, Tsai YT, Ke JY, Pan SH, Hsu YC, Li KC, Yang PC, Chen YL and Hong TM. alpha-Catulin drives metastasis by activating ILK and driving an alphavbeta3 integrin signaling axis. Cancer research. 2013; 73:428-438.

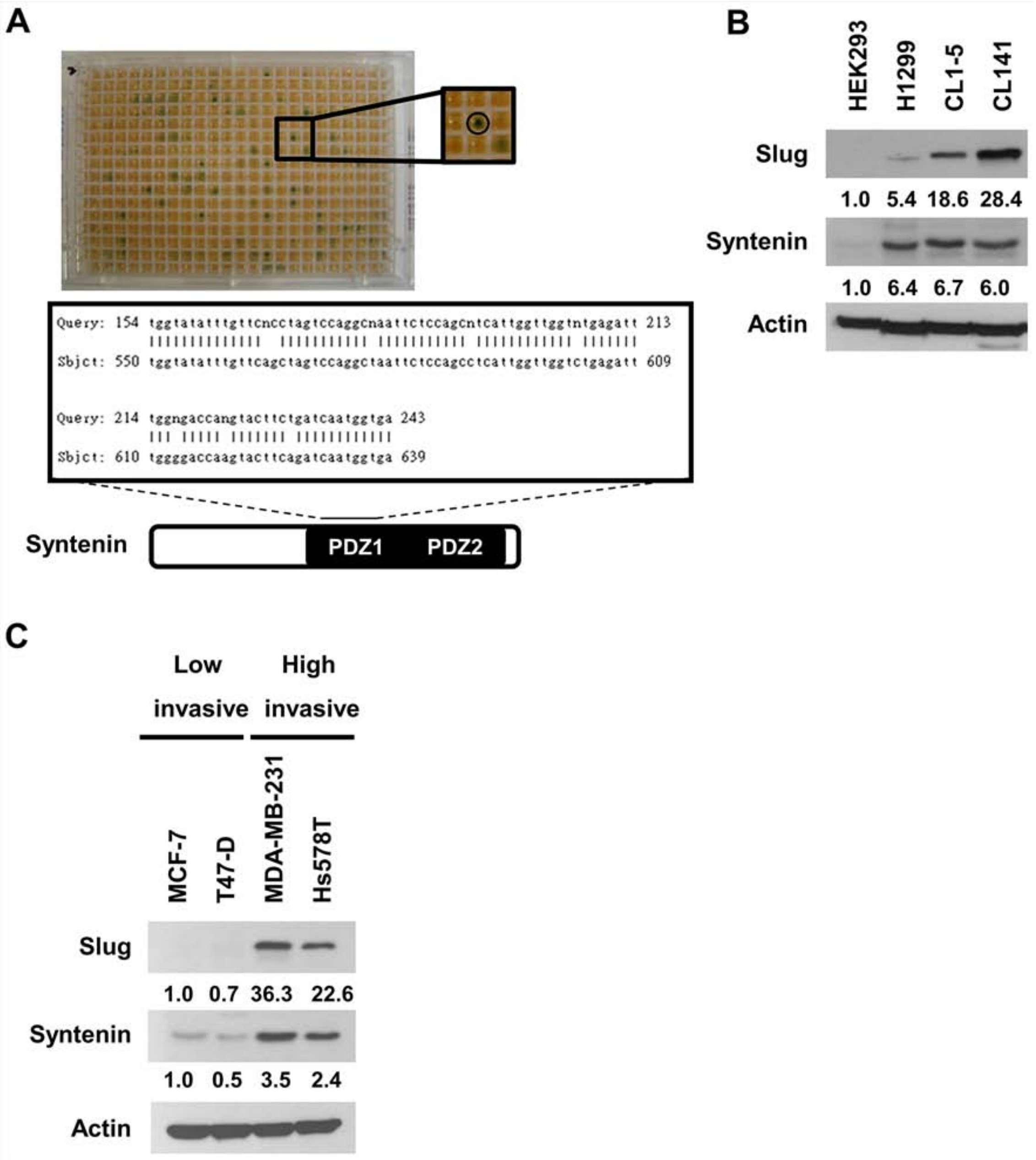

Supplementary Figure S1: MDA-9/Syntenin was a candidate of Slug-associating protein and both were highly expressed in high-invasive breast cancer cell lines. A. Yeast two-hybrid assay showed that the sequence of a Slug-associated candidate clone had 94% homology to the cDNA sequence of the PDZ1 domain of MDA-9/Syntenin. The yeast cells transformed with LexA-Slug were mated with yeast cells transformed pACT2 vector with specific genes from the human prostate cancer cDNA library. The mated yeast cells were then selected on a medium lacking histidine, leucine, and tryptophan. **B.** The expression level of MDA-9/Syntenin and Slug proteins in HEK293 and lung adenocarcinoma cell lines (H1299, CL1–5, and CL141) and **C.** breast cancer cell lines were analyzed directly by immunoblotting.

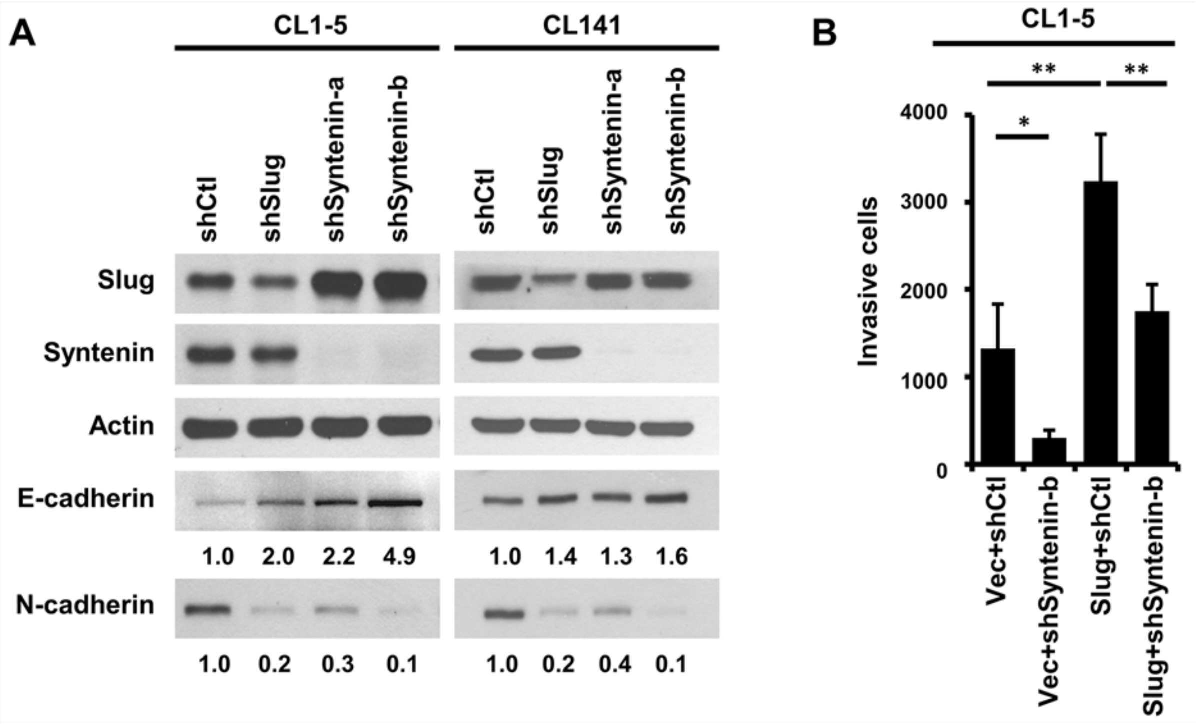

Supplementary Figure S2: MDA-9/Syntenin and Slug expressions positively correlated with the EMT process. **A.** CL1–5 (left) and CL141 (right) cells were infected with shSlug or shSyntenin viruses for 72 h and endogenous MDA-9/Syntenin, Slug, E-cadherin and N-cadherin were examined by immunoblotting. shLacZ was used as control. **B.** The invasive capacity of CL1–5/Vector+shCtl, CL1–5/Vector+shSyntenin-b, CL1–5/Slug+shCtl, and CL1–5/Slug+shSyntenin-b stable cells was determined with the Boyden chambers invasion assay.

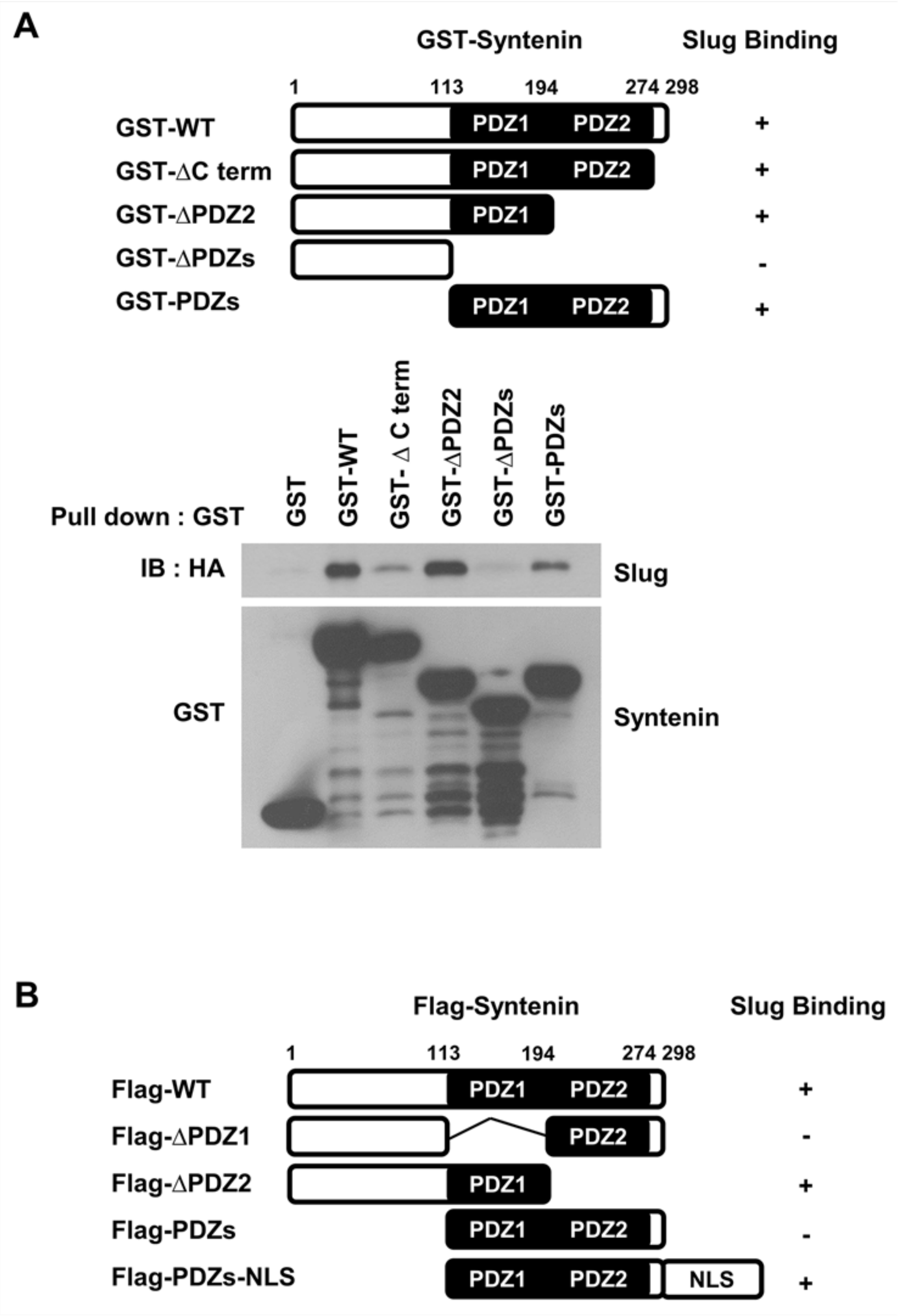

Supplementary Figure S3: The interaction between MDA-9/Syntenin deletion clones and Slug. A. The purified GST-fused deletion mutants of MDA-9/Syntenin were incubated with *in vitro* translated proteins of HA-tagged Slug and pulled down by glutathione beads. The associated Slug protein was determined by immunoblotting with anti-HA antibodies. **B.** Schematic graph representing the domain structure of Flag-Syntenin and its deletion mutants.

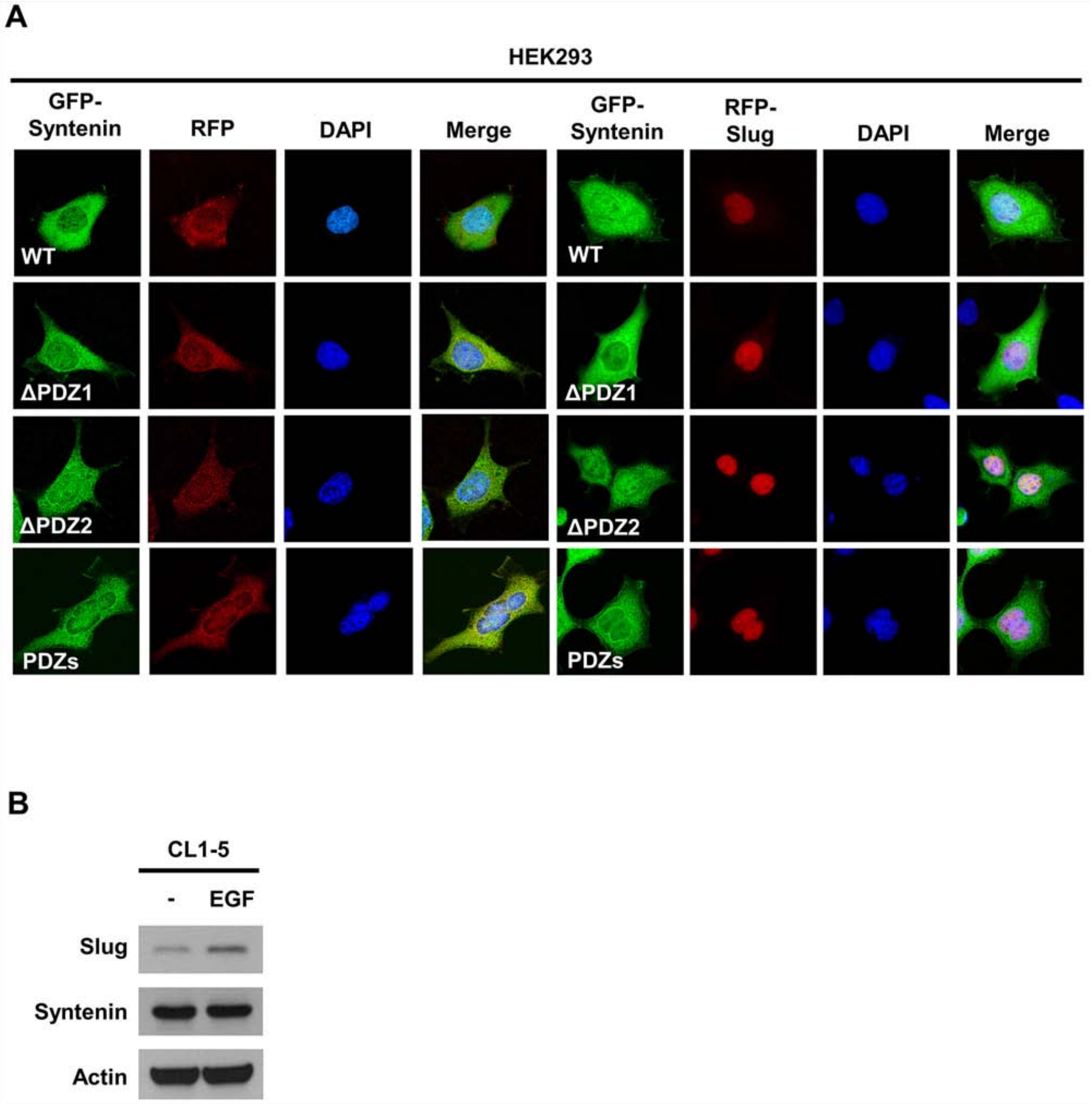

Supplementary Figure S4: The distribution of MDA-9/Syntenin mutants in HEK293 cells with or without Slug co-expression and EGF stimulation increased Slug protein expression. A. HEK293 cells were transiently co-transfected with plasmids expressing the indicated EGFP-Syntenin variants with DsRED-Slug for 14 h. The cells were then processed for DAPI nuclear staining. The cellular distribution of EGFP- Syntenin variants and DsRED-Slug were visualized by confocal microscopy. **B.** CL1–5 cells were cultured in serum-free medium for 12 h with or without EGF (40 ng/ml) treatment for 6 h, the lysates were examined by immunoblotting.

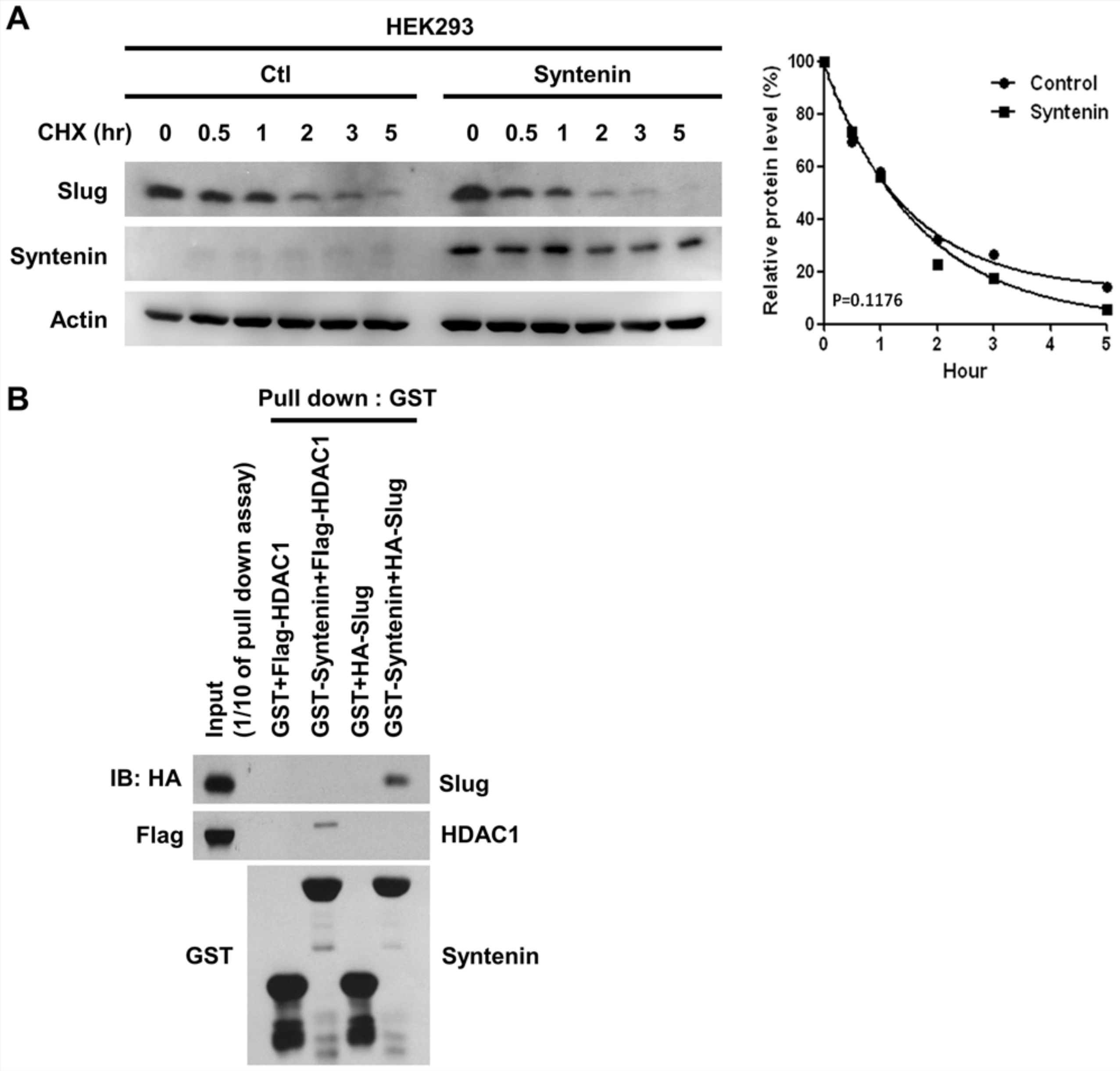

Supplementary Figure S5: MDA-9/Syntenin did not modulate Slug protein stability and associates with HDAC1. **A.** HEK293/Slug-expressed cells were transiently infected with control vector or MDA-9/Syntenin for 72 h. The cells were treated with cyclohexamide (100 µg/ml) for the indicated time points. Slug protein expressions were examined by Western blotting (left panel). The Slug band intensity was normalized to actin and the protein level of Slug were normalized to the control $t = 0$ (right panel). **B.** The indicated purified GST-Syntenin fusion proteins were incubated with *in vitro* translated proteins of HA-tagged Slug or HDAC1-Flag and pulled down by glutathione beads. The protein association was determined by immunoblotting.

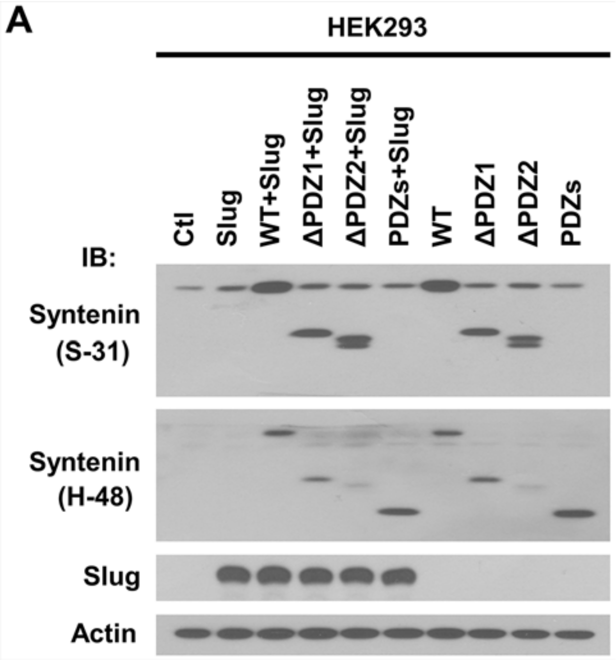

Supplementary Figure S6: The protein expression of MDA-9/Syntenin deletion constructs in Slug-expressing cells. **A.** HEK293 cells were infected with different viruses containing WT, ΔPDZ1, ΔPDZ2, and PDZs of MDA-9/Syntenin proteins with the control vector or Slug-expressing plasmid for 72 h. MDA-9/Syntenin and Slug protein expressions were examined by Western blotting. Anti-Syntenin antibodies (S-31) were used to identify the N-terminal region of MDA-9/Syntenin whereas anti-Syntenin antibodies (H-48) were used for the C-terminal region of MDA-9/Syntenin mutants.

Supplementary Table S1: Functional assay of MDA-9/Syntenin mutants to Slug-mediated regulation

	WT	ΔPDZ1	ΔPDZ2	PDZs	PDZs-NLS
**Interact with Slug**	+	-	+	+	ND
**Interact with Slug in cells**	+	-	+	-	+/-
**Accumulation in cell nucleus in Slug-expressing cells**	+	-	+	-	ND
**Enhancing Slug-mediated cell invasion**	+	-	-	-	+/-

Abbreviations: ND, not determined
Symbols: -, negative; +/-, weak; +, pronounced

Supplementary Table S2: The information of siRNA and shRNA

siRNA name	Sequence designation
siSyntenin	GCAAGACCUUCCAGUAUAA
siSlug	CCGUAUCUCUAUGAGAGUUACUCCA
**shRNA name**	**Target sequence of human genes**
shSyntenin-a (TRCN293258)	CCTATCCCTCACGATGGAAATCTC
shSyntenin-b (TRCN293259)	GAGAAGATTACCATGACCATTC
shSlug (TRCN15389)	CCCATTCTGATGTAAAGAAAT
shLacZ (TRCN72224)	CGCGATCGTAATCACCCGAGT

Supplementary Table S3: Primers used to amplify Slug, MDA-9/Syntenin, E-cadherin, Vimentin, Gβ-like

Gene Name	5′ primer designation	3′ primer designation
Slug	CATGCCATTGAAGCTGAAA AG	GCAGTGAGGGCAAGAAAAAG
MDA-9/ Syntenin	TTCTGCTCCTATCCCTCACG	AGAGCTCCATCCTGCACAGT
E-cadherin	GCTGGAGATTAATCCGGACA	ACCCACCTCTAAGGCCATCT-
Vimentin	GACAATGCGTCTCTGGCACGTCTT	TCCTCCGCCTCCTGCAAGTTCTT
Gβ-like	GTATGGAACCTGGCTAACTG	TACTGATAACTTCTTGCTTC
